# Grazing Management Regimen Shapes Soil Carbon Stocks and Associated Bacterial Community Composition in a Semi-Arid Rangeland

**DOI:** 10.3390/biology15201901

**Published:** 2026-10-21

**Authors:** Erickson Basave-Villalobos, José A. Sigala, María del Rosario Jacobo-Salcedo, Ignacio Sánchez-Cohen, Vicenta Constante-García, Judith Abigail Sánchez-Ledesma, Erika Nava-Reyna

**Affiliations:** 1 CIRPAS-Iguala Experiment Station, Instituto Nacional de Investigaciones Forestales, Agrícolas y Pecuarias, Iguala de la Independencia 40000, Guerrero, Mexico; basave.erickson@inifap.gob.mx; 2 CIRNOC-Valle del Guadiana Experiment Station, Instituto Nacional de Investigaciones Forestales, Agrícolas y Pecuarias, Durango 34170, Durango, Mexico; sigala.jose@inifap.gob.mx; 3 National Center of Disciplinary Research in Plant-Water-Soil-Atmosphere Relationship (CENID-RASPA), Instituto Nacional de Investigaciones Forestales, Agrícolas y Pecuarias (INIFAP), Gómez Palacio 35079, Durango, Mexico; jacobo.rosario@inifap.gob.mx (M.d.R.J.-S.); sanchez.ignacio@inifap.gob.mx (I.S.-C.); constante.garcia@inifap.gob.mx (V.C.-G.); 4 Tecnológico Nacional de Mexico, Instituto Tecnológico de Torreón, Torreón 27170, Coahuila, Mexico; judith.sl@torreon.tecnm.mx

**Keywords:** microbiome, grazing management, carbon stocks, rangeland resilience

## Abstract

Semi-arid rangelands are highly vulnerable to climate change and overgrazing, yet the functional coupling between soil carbon stabilization and microbial recovery remains largely unquantified. This study was carried out in Northern Mexico (24°0′33″ N, 104°42′28″ W, 1990 m.a.s.l.) and evaluated associations between a grazing management gradient comprising continuous grazing (CG), closed non-selective intensive grazing (GE), and open non-selective intensive grazing (SG) on soil carbon stocks and different labile fractions, bacterial community structures, and functional metabolic pathways in semi-arid soils. The results show that SG showed the highest observed organic and inorganic carbon stocks (SOC and SIC stocks, respectively), along with the labile carbon (LC) fraction. Conversely, GE favors the accumulation of stable, non-labile carbon (NLC). Although alpha diversity remained unaffected, grazing management induced differences in beta diversity. Redundancy and LEfSe analyses revealed a strong coupling between carbon fractions and microbial assembly, characterized by the exclusive enrichment of copiotrophic biomarker families (Burkholderiaceae and Sphingobacteriaceae) under SG. Furthermore, predictive functional profiling (PICRUSt2 and ANCOM) showed that pathways such as tRNA charging and nicotinate degradation were enriched under SG and GE, reflecting potential metabolic capacities and inferred microbial adjustments to heterogeneous, excreta-derived nutrient microenvironments during intermediate recovery states. Ultimately, the findings suggest that targeted rotational grazing may contribute to improved soil carbon stocks and microbial community recovery in semi-arid rangelands.

## Simple Summary

Drylands and natural grasslands are increasingly threatened by overgrazing and climate change, which damage soil functionality and reduce their natural carbon sequestration capacity. This study evaluated how different livestock management practices, such as high-stocking-density rotational grazing and temporary rest periods, relate to soil carbon storage and bacterial community structures. The findings suggest that non-selective intensive grazing is associated with higher soil carbon levels and distinct microbial groups linked to potential nutrient cycling and organic matter turnover. In contrast, long-term rest and fencing tend to favor more stable carbon forms. Ultimately, these exploratory results provide preliminary evidence that well-managed non-selective intensive grazing livestock practices may contribute to potential soil carbon storage and microbial community adjustments, supporting future restoration efforts in semi-arid rangelands.

## 1. Introduction

Livestock grazing represents a widespread land-use practice with profound economic and environmental implications. Growing evidence links grazing management to greenhouse gas emissions and broader climate change dynamics, primarily through its direct effects on vegetation litter inputs, soil aeration, and manure deposition [1,2,3]. Moreover, rangeland and shrubland degradation in drylands is driven primarily by overgrazing and climate change [4]. In contrast, management strategies such as rotational and moderate grazing can improve soil organic carbon stock (SOC stock), structural stability, and bacterial diversity compared to continuous or heavy grazing [5,6,7]. Even regenerative grazing practices, such as non-selective intensive grazing, are purported to sequester greater amounts of carbon (C) in grassland soils than rotational grazing at low stocking densities [6,8]. These benefits are mediated by plant–microbial–nutrient feedbacks that enhance ecosystem resilience to disturbance [9]. Because microorganisms act as fundamental catalysts of global biogeochemical cycles, elucidating the molecular mechanisms underlying microbe-mediated functions is essential for sustainable grassland management [2]. Furthermore, bacterial diversity, network complexity, and stability enhance soil ecosystem multifunctionality, while shifts in bacterial composition can weaken it [10,11,12]. Network complexity and stability are more robust predictors of shifts in ecosystem multifunctionality than species diversity on its own [9].

Specific management practices further reinforce these dynamics. Some grazing management regimes, such as moderate grazing, spring rest-grazing, multi-species livestock integration, and high stocking density rotational systems, improve soil physical structure, elevate particulate and mineral-associated organic carbon, and alleviate microbial stress by enhancing organic matter and plant diversity [6,9,13,14]. However, while these sustainable practices mitigate degradation, soils in semi-arid and rangelands are simultaneously subjected to severe compounding abiotic constraints; in particular, secondary salinization, osmotic pressure, sharp pH fluctuations, and variations in salt or gypsum accumulation exacerbate environmental harshness, intensifying metabolic limitations and driving shifts in bacterial community structure [15,16].

In arid and semi-arid shrublands, the benefits of grazing coexist with severe edaphoclimatic constraints, such as aridity, osmotic stress, sharp pH fluctuations, and variations in salt or gypsum accumulation [16,17,18]. Factors such as water availability and physicochemical properties often decouple or strongly condition how bacterial communities respond to vegetation attributes [17,19]. Indeed, under high pressure or pronounced aridity, bacterial communities must restructure toward stress-tolerant taxa [15,16]. Furthermore, structural equation models determine that aridity and hydrophysical climatic variables directly constrain microbial productivity and functioning [18,20].

While previous studies in rangelands have separately addressed either grazing management impacts on soil organic carbon pools or microbial taxonomic shifts, the integration between labile carbon fractions and the inferred functional metabolic potential of soil microbiomes remains largely unquantified, particularly in arid lands. Specifically, it remains poorly understood how grazing rotation drives the stoichiometric coupling between carbon stabilization and microbial metabolic profiles.

We hypothesized that grazing management regimes act as selective environmental filters linked with variations in carbon stoichiometry and the composition, diversity, and inferred metabolic functions of soil bacterial communities. Thus, this work aimed to evaluate the possible effect of a grazing management gradient on the dynamics of soil organic carbon fractions and the composition and functional metabolic structure of bacterial communities, determining how these responses reflect ecosystem recovery in semi-arid rangeland.

## 2. Materials and Methods

### 2.1. Study Site Description and Sampling

The research was structured to evaluate broad, community-level contrasts across long-term grazing regimes in the ejido Lazaro Cárdenas, located in the Municipality of Durango, Durango, Mexico (Figure 1). The experimental area (24°0′33″ N, 104°42′28″ W) is located on a south-facing slope with a 15% inclination, at an elevation of 1990 m above sea level (m.a.s.l.). The region has a semi-dry temperate climate, characterized by a mean annual temperature of 17 °C and a total annual precipitation of 475 mm. Pedologically, the soils are very shallow (less than 25 cm deep) and stony. The baseline physicochemical properties of the study sites are detailed in Table S1.

This study evaluated a pre-existing management gradient across contiguous ejido zones in Durango maintained over 15 years under three distinct regimes: (1) closed non-selective intensive grazing (GE), characterized by short-duration, high-density management (1 ha/AU), rotating every 3–4 h until forage depletion followed by an 18-month rest period; (2) continuous grazing (CG), defined as traditional grazing practiced on regional ejido lands since the last century, operating at an average stocking rate of 3.5 ha/AU under continuous pressure; and (3) open non-selective intensive grazing (SG)—an intermediate condition comprising an area managed under GE that was subsequently reopened to grazing 6 months prior to sampling. Grazing across the managed areas was historically sustained by domestic livestock (primarily creole cattle and, to a lesser extent, equines).

To capture local micro-environmental heterogeneity and ensure regional representativeness within each contiguous, community-managed zone, three spatial quadrats of 400 m^2^ separated by approximately 50 to 100 m were established within the same paddock. Sites were selected aiming for similarity in south-facing slope, parent lithology, historical slope gradients, and native vegetation assemblages dominated by shrublands and grasslands adapted to semi-arid conditions (*Acacia schaffneri*, *Neltuma laevigata*, *Opuntia durangensis*, and *Bouteloua* sp.) with varying understory grass cover, which remained comparable across the study areas. Vegetation cover and above-ground biomass reflected the degradation gradient, showing higher structural complexity and litter accumulation in GE and SG compared to the heavily depleted cover observed in CG.

For carbon stock analysis, four surface soil subsamples (0–20 cm depth) were randomly collected from each spatial quadrant and thoroughly homogenized to form a single composite sample per quadrant, yielding three spatial replicates (*n* = 3) per treatment. For bacterial community analysis, a soil sample from each of the three quadrants per treatment was processed by in situ cell disruption: 0.25 g of the collected soil was placed into Zymo Research BashingBead™ (Zymo Research, Irvine, CA, USA) tubes containing 750 µL of lysis solution and processed in a TerraLyzer™ cell disruptor (Zymo Research, Irvine, CA, USA) for 30 s. Samples were then cryopreserved prior to laboratory-based DNA extraction.

Finally, intact clods of approximately 50–100 cm^3^ were collected from each quadrant to determine bulk density (BD) using the paraffin-coated clod method [21].

### 2.2. Analysis of Carbon Stocks and Carbon Fractions

All carbon stocks and fractions were analyzed in individual composite samples of each quadrant. Soil organic matter (SOM) and soil organic carbon (SOC) contents were determined following the Walkley and Black [22] wet oxidation method, whereas soil inorganic carbon (SIC) was quantified as carbonate content via gas volumetry using a Bernard calcimeter [23] on each composite sample. Total soil organic carbon (SOC stock) and total soil inorganic carbon (SIC stock) stocks (Mg C ha^−1^) were calculated using the following equation [24]:

SOC (SIC)stock = SOC(SIC) × BD × D × 10

where the concentration of SOC or SIC is in g C kg^−1^ of dry soil, BD is the bulk density (Mg m^−3^), D is the soil depth (0.2 m), and 10 is a conversion factor.

Total carbon (TC) content in the analyzed soils was determined via dry combustion using an elemental analyzer (TOC-L, Shimadzu, Kyoto, Japan). The quantification of carbon fractions with varying degrees of lability was performed following the method established by Blair et al. [25], which relies on the degree of carbon oxidation using a 0.333 M potassium permanganate (KMnO_4_) solution. Results were expressed in grams of carbon per kilogram of dry soil (g C kg^−1^), where the labile carbon (LC) fraction corresponded to the portion oxidized by the permanganate solution, and the non-labile (NLC) fraction was calculated as the remainder of the total carbon.

### 2.3. DNA Extraction and the 16S rRNA Gene Amplification

Bacterial DNA was extracted from the soil samples using the ZymoBIOMICS™ DNA Miniprep Kit (Zymo Research, Irvine, CA, USA). Subsequently, DNA purity and concentration were quantified on a Thermo Scientific™ Multiskan SkyHigh™ microplate spectrophotometer (Thermo Fisher Scientific, Waltham, MA, USA) equipped with a µDrop™ microplate (Thermo Fisher Scientific, Waltham, MA, USA).

Bacterial 16S rRNA gene amplification targeted the V3-V4 hypervariable region using the primers established by Klindworth [26]—S-D-Bact-0341-bS-17, 5′-CCTACGGGNGGCWGCAG-3′ and S-D-Bact-0785-a-A-21, 5′-GACTACHVGGGTATCTAATCC-3′—which yield an approximate ~460 bp amplicon. Primers were modified to incorporate Illumina overhang adapters [27,28], resulting in the following full sequences, with an expected initial amplicon length of ~550 bp: 5′–TCGTCGGCAGCGTCAGATGTGTATAAGAGACAGCCTACGGGNGGCWGCAG–3′ and 5′–GTCTCGTGGGCTCGGAGATGTGTATAAGAGACAGGACTACHVGGGTATCTAATCC–3′.

Polymerase chain reactions (PCRs) were conducted following standard Illumina protocols [28] in a Labnet Multigene™ Gradient PCR thermocycler (Labnet International Inc., Edison, NJ, USA). Each 25 µL reaction mixture contained 12.5 µL of 1X MyTaq™ Ready Mix (Bioline^®^, London, UK), 1 µL of each primer (10 µM), 5 µL of template DNA (~50 ng), and 5.5 µL of molecular-grade water. Thermal cycling conditions consisted of an initial denaturation at 95 °C for 3 min; 25 cycles of 95 °C for 30 s, 55 °C for 30 s, and 72 °C for 30 s; and a final extension step at 72 °C for 5 min. Amplicon size (~550 bp) and quality were verified by analyzing 1 µL of PCR product on a Bioanalyzer DNA 1000 chip (Agilent Technologies, Santa Clara, CA, USA), and products were subsequently cleaned using 0.8% Agencourt^®^ AMPure^®^ XP beads (Agilent Technologies, Santa Clara, CA, USA).

For multiplex sequencing preparation, indexing was performed using the Nextera XT Index Kit™ (Illumina, San Diego, CA, USA) [27]. The index PCR mix contained 25 µL of 1X MyTaq™ Ready Mix (Bioline^®^, London, UK)) and 5 µL of each indexing primer (N7xx and S5xx), cycled under the following profile: initial denaturation at 95 °C for 3 min; 10 cycles of 95 °C for 30 s, 55 °C for 30 s, and 72 °C for 30 s; and a final extension at 72 °C for 5 min. Libraries were purified using 1.2% Agencourt^®^ AMPure^®^ XP beads Agencourt^®^ AMPure^®^ XP beads (Agilent Technologies, Santa Clara, CA, USA), and the expected final library size (~630 bp) was confirmed via Bioanalyzer DNA 1000 chip Agencourt^®^ AMPure^®^ XP beads (Agilent Technologies, Santa Clara, CA, USA) analysis on randomly selected samples. Finally, libraries were quantified, normalized for equimolar pooling, and sequenced on an Illumina^®^ MiSeq platform (Illumina, San Diego, CA, USA) (2 × 250 bp paired-end configuration) according to the 16S Metagenomic Sequencing Library Preparation Protocol [28].

### 2.4. Bioinformatic Analysis

Sequencing data processing was performed within a Linux–Ubuntu environment using QIIME2 2023.5 [29]. The bioinformatic analysis scripts and workflow protocols for 16S rRNA gene amplicon sequencing supporting the findings of this study are openly available on GitHub at https://github.com/erinava27/QIIME2-Workflow-for-Soil-Bacterial-Community-Analysis.git (accessed on 15 October 2026). Quality control, error filtering, chimera filtration, and amplicon sequence variant (ASV) inference were executed via DADA2 [30,31] using the qiime dada2 denoise-paired plugin. Rather than fixed-position truncation (--p-trunc-len-f 0, --p-trunc-len-r 0), low-quality tails and erroneous reads were dynamically filtered using a truncation quality threshold (--p-trunc-q 5) and maximum expected error limits (--p-max-ee-f 2, --p-max-ee-r 4). Taxonomic classification was subsequently assigned against the Greengenes2 reference database (2022) [32]. Prior to diversity analyses and taxonomic richness counts, sequencing depth was rarefied to an even sampling depth of 30,000 sequences per sample. This depth was selected based on the rarefaction plateau (Figure S1) to maximize the retention of high-quality samples while ensuring adequate coverage of bacterial taxonomic richness. ASV species-level assignments were cross-validated against the NCBI database using BLAST ((version v2.12.0; applying strict thresholds of 100% query coverage, E-value ≤ 0.001, and ≥98% sequence identity). Relative abundances of dominant taxa were visualized using the Morpheus—Broad Institute webserver (https://software.broadinstitute.org/morpheus (accessed on 3 June 2026).

Alpha diversity was evaluated using four indices—observed ASVs, Shannon diversity, Pielou’s evenness, and Faith’s phylogenetic diversity—where taxonomic richness metrics were calculated at the sample level using the rarefied feature table, representing unique amplicon sequence variants detected within each independent composite sample per treatment. Group differences were tested via the Kruskal–Wallis test (*p* < 0.05). For beta diversity, Jaccard, Bray–Curtis, unweighted UniFrac, and weighted UniFrac dissimilarities were calculated. Community clustering patterns were visualized through Principal Coordinates Analysis (PCoA) via the Emperor plugin [33], and statistical significance across treatments was determined using Permutational Multivariate Analysis of Variance (PERMANOVA) with 999 permutations.

Bacterial functional profiles were predicted using PICRUSt2 (v2.5.2) [34]. QIIME2-generated ASVs were aligned against reference genomes from IMG/M and KEGG Orthology databases utilizing HMMER and EPA-NG, with phylogenetic placements processed via GAPPA [35,36,37,38,39]. Gene family abundances were normalized by 16S rRNA gene copy numbers, and prediction reliability was assessed using the Nearest Sequenced Taxon Index (NSTI). Metabolic pathway abundances were extracted from the MetaCyc database [40] (via the file path_abun_unstrat.tsv.gz). Prior to differential abundance testing, metabolic pathway tables were filtered to retain features present in at least 2 samples with a minimum frequency threshold of 10 (--p-min-samples 2, --p-min-frequency 10). To handle zero-abundance values inherent to compositional data, a pseudocount of 1 was added (qiime composition add-pseudocount). Differential abundance across grazing management groups was assessed using qiime composition ANCOM, where the resulting W statistic denotes the number of pairwise comparisons in which a given metabolic pathway successfully rejects the null hypothesis of equal relative abundance. Downstream functional compositions and differential pathways were visualized in R version 4.4.3 (28 February 2025 ucrt) within RStudio version 2025.05 [41,42].

The LEfSe analysis was also performed in RStudio using the microeco package (v.1.x). The statistical workflow implemented a non-parametric factorial Kruskal–Wallis sum-rank test among grazing treatments with an alpha significance threshold of *p* < 0.05, followed by pairwise Wilcoxon rank-sum tests to assess biological consistency among subclasses. A multi-class strategy (“all-against-all”) was employed for comparisons across the three management systems. As standard for the classic LEfSe algorithm, no Benjamini–Hochberg false discovery rate (FDR) correction was applied to the initial Kruskal–Wallis step, relying instead on a logarithmic LDA score threshold greater than 3.0 as a strict effect-size filter of robustness.

### 2.5. Data Processing and Statistical Analysis

Prior to statistical evaluations, carbon content datasets were assessed for normality and homogeneity of variance. Since all carbon stocks and fractions failed to meet these parametric assumptions, data were transformed using a log_10_ function prior to analysis. Significant differences among management treatments were subsequently determined using one-way analysis of variance (ANOVA) followed by Tukey’s post hoc test (*p* < 0.05). To explore relationships among dominant bacterial phyla and environmental parameters across soil conditions, a Principal Component Analysis (PCA) was performed in RStudio 2025.05.1 to identify key variables driving microbial patterns. Furthermore, Redundancy Analysis (RDA) was executed to evaluate multivariate relationships and patterns linking grazing management with carbon stocks utilizing the “vegan” package in RStudio [43].

## 3. Results

### 3.1. Evaluation of Carbon Stocks and Labile Pools

As shown in Figure 2, grazing management significantly affected both carbon stocks (F_(2,6)_ = 29.08, *p* ˂ 0.001, and F_(2,6)_ = 6.313, *p* = 0.033, for SIC and SOC stocks, respectively) and carbon fractions (F_(2,6)_ = 9.777, *p* = 0.013, and F_(2,6)_ = 17.094, *p* = 0.003, for LC and NLC, respectively). Specifically, SIC and SOC stocks were highest under SG, followed by CG for SIC and GE for SOC. Regarding carbon lability, LC content was significantly higher in SG and CG, whereas NLC predominated under GE.

In addition, Principal Component Analysis (PCA) revealed distinct patterns among grazing management regimes (Figure 3). The loading plot revealed the associations among the carbon fractions, indicating that the variables were effectively captured within the first two principal components.

The first two principal components accounted for 90.60% of the total variance. Furthermore, sample groupings showed clear spatial separation and clustering according to management type. The PCA ordination showed clear environmental segregation determined by carbon dynamics. Specifically, open non-selective intensive grazing management is closely aligned with the soil labile carbon fraction, followed by continuous grazing. Conversely, closed non-selective intensive grazing samples were ordinated in the direction of variables representing non-labile and stable carbon stocks. Overall, the PCA plot also shows that both closed and open non-selective intensive grazing increase SOC stocks. These multivariate patterns reinforce the notion that targeted grazing strategies are associated with variations in soil carbon storage and fraction distribution, reflecting distinct environmental configurations across management regimes.

### 3.2. Shifts in Soil Microbial Communities Across Grazing Management Regimes

The overall mean number of sequences obtained across all treatments was 206,999, with an average of 160,901 successfully merged sequences (Table S2). Rarefaction curves approached a plateau, supporting the view that sequencing depth was adequate for the observed richness and indicating that the sequencing effort was sufficient to capture the majority of phylum-level bacterial taxonomic diversity present in the samples (Figure S1).

According to Figure 4, no influence of grazing management was detected in alpha diversity.

As illustrated in Figure 5, beta diversity differed significantly among grazing treatments based on the Jaccard distance (presence/absence-based), Bray–Curtis dissimilarity (abundance-based), and unweighted UniFrac (phylogenetic lineage presence/absence). In contrast, weighted UniFrac (incorporating both phylogeny and relative abundance) showed no significant differences among management regimes.

Multivariate homogeneity of group dispersions (PERMDISP) was tested to verify whether significant differences detected by PERMANOVA were influenced by dispersion heterogeneity. The PERMDISP analysis revealed no significant differences in multivariate dispersion among the grazing regimes for Bray–Curtis (F_(2,6)_ = 0.9556, *p* = 0.521), Jaccard (F_(2,6)_ = 2.3018, *p* = 0.201), unweighted UniFrac (F_(2,6)_ = 1.6177, *p* = 0.291), and weighted UniFrac (F_(2,6)_ = 1.4584, *p* = 0.411). These non-significant results confirm that group dispersions were homogeneous across treatments, indicating that community structural variations detected by PERMANOVA reflect true compositional shifts (location changes) rather than artifacts of dispersion heterogeneity.

Taxonomic profiling based on sequencing data revealed distinct richness patterns across management systems. Specifically, the GE treatment harbored 35 phyla, 83 classes, 235 orders, 401 families, 742 genera, and 967 species. The SG treatment comprised 34 phyla, 74 classes, 229 orders, 399 families, 778 genera, and 1042 species, whereas the CG treatment exhibited 34 phyla, 74 classes, 104 orders, 342 families, 614 genera, and 761 species. Raw sequencing reads were deposited in the National Center for Biotechnology Information (NCBI) Sequence Read Archive under BioProject accession number PRJNA1520797.

According to phylum-level taxonomic composition (Figure 6), Actinobacteriota and Acidobacteriota were the dominant phyla across all grazing management regimes. Nevertheless, Actinobacteriota showed higher relative abundance in CG, followed by SG, whereas Proteobacteria was most abundant in SG, followed by GE.

Moreover, LefSe analysis at the family level showed significant taxonomic enrichment exclusively associated with the SG treatment. Using a stringent logarithmic LDA score threshold of 3.0, two bacterial families (Burkholderiaceae and Sphingobacteriaceae) were identified as primary biomarkers significantly enriched in SG soils (Figure 7). Furthermore, using a standard threshold of LDA > 2.0, a total of ten families showed significant differential abundance in SG management (Kruskal–Wallis, *p* < 0.05) (Figure S2).

### 3.3. Relationship Between Soil Bacteria Communities and Carbon Stocks and Fractions

The Redundancy Analysis (RDA) highlighted how different carbon pools are linked to microbial distribution across grazing management regimes (Figure 8). The ordination suggests an ecological differentiation associated with carbon lability and stock types. The ordination revealed that specific bacterial taxa are closely coupled with distinct carbon pools. Particularly, Actinobacteriota aligned strongly with LC and soil SIC stock, underscoring their role as dynamic copiotrophs driving rapid carbon turnover. In contrast, taxa such as Acidobacteria seem to respond negatively to higher gradients of those carbon fractions and stocks. Moreover, Proteobacteria, Bacteroidota, and Firmicutes showed closer associations with SOC stock and NLC. This multivariate coupling provides preliminary evidence that grazing-induced shifts in soil carbon pools act as a strong environmental filter, selectively shaping bacterial community functions and metabolic pathways to sustain ecosystem resilience.

### 3.4. Response of Inferred Metabolic Pathways to Grazing Management Practices

Functional predictions using PICRUSt2 identified a total of 417 predicted metabolic pathways within the grazing management regimes. The accuracy of the functional predictions was evaluated using the weighted Nearest-Sequenced Taxon Index (NSTI). The mean weighted NSTI value across samples was 0.143 ± 0.018, which falls well within the acceptable threshold for complex environmental samples such as soils, confirming the reliability of the predicted metabolic pathways (Table S2).

The functional metabolic profile is illustrated in Figure 9, showing that the most abundant pathways correspond to aerobic respiration I (cytochrome c) ($\bar{x}$ = 1.98%), pyruvate fermentation into isobutanol ($\bar{x}$ = 1.25%), cis-vaccenate biosynthesis ($\bar{x}$ = 1.07%), L-isoleucine biosynthesis ($\bar{x}$ = 1.07%), and L-valine biosynthesis ($\bar{x}$ = 1.07%) (Figure 9).

According to the ANCOM, the predicted pathways for tRNA charging (TRNA-CHARGING-PWY, W = 52) and nicotinate degradation I (PWY-722) (W = 12) emerged as the most robust differentially abundant features among treatments, reflecting the inferred metabolic adaptations of the soil bacterial communities across the evaluated grazing systems (Figure 10). Specifically, these predicted pathways were found to be enriched in the GE and SG regimes and SG management alone (Table S3).

## 4. Discussion

Consistent with our hypothesis, the results showed that different livestock management practices serve as environmental filters, governing carbon stoichiometry and directing both the taxonomic composition and metabolic capabilities of soil bacteria in semi-arid environments.

### 4.1. Carbon Storage and Labile Pool Dynamics

The carbon dynamics observed in this research provide insight into how grazing management is associated with variations in carbon storage in the study region.

The significant enhancement in SOC stocks and labile fraction under open non-selective intensive grazing (SG) suggests that well-designed management may contribute to potential carbon storage dynamics and, consequently, support environmental recovery trajectories in semi-arid environments. Previous research corroborates that controlled rotational systems promote soil carbon accumulation and alter fraction dynamics, whereas livestock directly enhances short-term, labile carbon pools, including particulate organic carbon, hot water extractable carbon, and light fraction organic carbon [5,44,45]. The meta-analysis by McDonald et al. [46] found higher SOC stocks under rotational grazing compared with continuous grazing. Moreover, Mosier et al. [45] reported that adaptive multi-paddock (AMP) grazing sites averaged 13% more soil C than conventional grazing sites, mainly in the mineral-associated organic matter fraction. Other researchers have reported that non-selective grazing drives this pattern through severe herd impact and rapid defoliation [6,8]. The mechanical treading of livestock integrates dense standing litter directly into the soil mineral matrix, thereby shielding it from photo-oxidation [8,47]. Furthermore, previous research has reported that intensive grazing pressures drive uniform forage removal and induce pulsed root turnover, which fuel soil microbial activity and produce persistent microbial necromass stabilized on fine mineral surfaces [6,48]. Simultaneously, rapid and intensive defoliation fosters macro-aggregate formation, protecting otherwise labile fractions from enzymatic degradation [8]. Additionally, the continuous input of animal excreta deposits decomposable and dissolved carbon compounds [44].

Overgrazing, in contrast, disrupts the transformation of organic substrates into stable carbon forms by impairing microbial functionality and enzymatic processes, causing parallel declines in both POC and MAOC sub-pools [49]. This disruption could explain the lower SOC stocks observed in the continuous grazing area, where the balance between labile inputs from animal excreta and stable carbon formation is disrupted under heavy continuous pressure [50].

On the other hand, closed non-selective intensive grazing influences the non-labile carbon fraction, showing the importance of good management practices in conserving stable carbon stocks in soils. According to Abagandura et al. [8], closed high-stocking-density rotational grazing increases deep root growth and fungal network development by incorporating extended recovery intervals, which are critical for macro-aggregate formation and protect organic components from rapid enzymatic degradation. In addition, undisturbed microbial communities enhance the stabilization of chemically recalcitrant derivatives by binding to silt and clay particles in mineral-associated organic matter (MAOM) [8,51]. Likewise, the persistence of deep roots during resting phases enhances subsurface organic carbon stabilization while protecting the soil against the wind and water erosion typically observed in continuously grazed systems [44]. Notably, the Principal Component Analysis ordination plot illustrates a clear association of both closed and open non-selective intensive grazing with enhanced SOC stocks, which may be related to the preservation of above- and below-ground biomass due to optimized stocking schedules. Additionally, rotational rest periods increase moisture retention, protecting surface soils from drying, high compaction, and carbon leaching [44,52,53]. Soils with higher carbon stocks better buffer thermal and hydric stress for rangeland vegetation, directly benefiting plant nutrition and subsequently enhancing pasture productivity without environmental degradation [6,54,55].

Otherwise, soil inorganic carbon stocks are mainly related to pedogenic and lithogenic calcium carbonates, representing the largest carbon pool in arid and semi-arid soils [56,57]. Unlike dynamic organic carbon pools, SIC stocks respond primarily to parent mineralogy, soil depth, and long-term rainfall gradients rather than short-term livestock movements or management cycles [56,58]. In our study, carbonate concentrations exhibited spatial variation across treatments, a pattern consistent with the inherent vertical and lateral distribution of carbonates. While long-term exclusion or intensive management can theoretically modulate carbonate dynamics, for instance, through root respiration and associated carbonic acid production under intensive grazing that may accelerate carbonate dissolution [57,58], fundamental physical soil properties and moisture regimes impose strict boundaries on carbonate storage, effectively dampening grazing-induced shifts [57,58,59]. Thus, the variation in SIC stock may be an intrinsic site covariate reflecting background pedological and geomorphological heterogeneity rather than a direct outcome of the grazing regimes. Furthermore, the PCA corroborates this interpretation, demonstrating that the vector for soil inorganic carbon stock projects independently of the major management groupings driven by organic carbon fractions (Figure 3). This spatial decoupling further reinforces that SIC variation is governed by background pedological attributes rather than direct grazing-induced effects.

### 4.2. Restructuring of Soil Bacterial Communities and Optimization of Carbon Pools Under Varying Grazing Management Regimes.

Grazing management did not significantly alter bacterial alpha diversity; however, it influenced beta diversity across Jaccard, Bray–Curtis, and unweighted UniFrac metrics (Figure 4 and Figure 5). Similar findings have been reported by other authors. For instance, a global meta-analysis on the Qinghai–Tibetan Plateau by Jing et al. [60] demonstrated that grazing alters soil bacterial and fungal community compositions while affecting beta diversity without changing alpha diversity. Yang et al. [61] observed a comparable response in a Tibetan alpine meadow. Furthermore, the significant variation detected in unweighted UniFrac, in contrast to the lack of significance in weighted UniFrac, suggests that grazing regimes primarily alter the presence or absence of rare phylogenetic lineages rather than causing proportional shifts in the most abundant taxa.

Regarding the most abundant phyla, Actinobacteriota, Proteobacteria, and Acidobacteriota were dominant across treatments. These phyla are universally dominant in soils, playing fundamental roles in nutrient cycling and organic matter decomposition [62,63,64,65,66]. Members of Actinobacteriota often exhibit plant growth-promoting properties, supplying nutrients to host plants and producing phytohormones to help cope with biotic stress induced by phytopathogens. Furthermore, they participate in organic matter mineralization and are capable of surviving under extreme conditions, including high salinity [67]. Owing to their r-strategist behavior, they are well suited to rapidly exploiting fresh organic matter inputs (such as urine, manure, and plant residues derived from grazing) [3,68]. Overgrazing has also been reported to increase the abundance of this phylum [69]. In contrast, members of the phylum Acidobacteria are K-strategists that grow more slowly in nutrient-limited soils [70]. This phylum has been documented to decrease in abundance in soils disturbed by excess nutrients resulting from intensive grazing [71]. Consequently, continuous grazing shifts the community from slow-growing oligotrophs, such as Acidobacteria, toward fast-growing copiotrophs, such as Actinobacteriota, thereby altering phylogenetic lineage presence without uniformly changing dominant abundance ratios, as previously reported by Xun et al. [72]. These patterns align with the RDA results (Figure 8), indicating an association between stable carbon pools and specific bacterial populations potentially involved in the utilization of persistent organic substrates. These patterns suggest a close link between soil carbon dynamics and microbial community assembly associated with different grazing practices.

LEfSe analysis at the family level also showed that Burkholderiaceae and Sphingobacteriaceae were primary biomarkers significantly enriched in SG soils (Figure 7). Members of the family Burkholderiaceae belong to the phylum Proteobacteria and are fast-growing copiotrophs that thrive in nutrient-rich rhizosphere soils, and their members also play a central role in plant adaptation in arid soils [73]. Additionally, members of the family Sphingobacteriaceae belong to the phylum Bacteroidota, which comprises copiotrophic decomposers closely associated with labile carbon turnover [74,75]. The relative abundance of these copiotrophic taxa aligns with the availability of accessible organic compounds originating from livestock excreta and fresh vegetation. Conversely, the NLC accumulation under GE coincides with an expansion of slow-growing, oligotrophic groups like Acidobacteria, which are evolutionarily tailored to degrade recalcitrant carbon in low-disturbance environments [76,77]. This taxonomic divergence highlights how different management regimes correspond to distinct microbial community structures, with continuous and non-selective intensive grazing favoring contrasting bacterial biomarker profiles. Rotational grazing at high stocking density showed an intermediate disturbance that enhances beta diversity and enriches copiotrophic biomarker families without triggering the community homogenization typically observed under heavy grazing. Overall, these patterns suggest that grazing management influences soil microbial composition by altering local resource availability, reinforcing the importance of tracking microbial indicators alongside soil biochemical properties.

The exclusive concentration of these biomarker families within the SG group aligns closely with genus-level observations (Figure S3), suggesting a distinct compositional shift associated with this management practice rather than a taxonomic-level artifact. Furthermore, these exploratory results provide preliminary evidence that improved grassland management can enhance soil organic carbon stocks, potentially supporting climate change mitigation and adaptation, an outcome of paramount importance for semi-arid rangelands vulnerable to global environmental change [2,78].

### 4.3. Inference of Functional Metabolic Responses of Soil Bacterial Communities to Grazing Practices

Grazing management restructures soil microbial communities, shifting dominant metabolic pathways from aerobic respiration to fermentation and altering amino acid and vitamin metabolism [2,78]. These patterns coincide with the higher abundance of the pyruvate fermentation to isobutanol pathway under SG, as well as isoleucine and L-valine biosynthesis under CG in this study (Figure 9), which may represent a stress-response signature rather than strictly a growth or biosynthesis pathway [79]. These functional adaptations appear to reflect distinct nutrient cycling strategies under different grazing intensity regimes [1,80].

The enrichment of the tRNA-charging pathway under open non-selective intensive grazing management could suggest that rotational nutrient pulses foster a metabolically prepared community capable of rapid peptide chain elongation and protein synthesis when excreta-derived resources become available under favorable soil conditions, such as moisture, organic matter, and reduced compaction, under rotational management [81,82]. However, because PICRUSt2 reflects gene content potential rather than direct transcriptional activity, this inference remains a predictive metabolic hypothesis that requires validation.

Furthermore, the predicted enrichment of the nicotinate degradation pathway (PWY-722) under SG management could be related to the microbial utilization of vitamin B3 derivatives as carbon and nitrogen sources [83,84]. Given that SG systems experienced a rotational opening period prior to sampling, this metabolic signature likely reflects spatial heterogeneity in soil nutrients. Because livestock return over 80% of consumed nitrogen via excreta and urine, rotational patches create transient, nutrient-rich microenvironments [85,86]. Consequently, the inferred upregulation of nicotinate degradation serves as a potential indicator of bacterial communities rapidly exploiting these localized, excreta-derived resources during an intermediate recovery state.

While predictive functional profiling offers valuable insights into the metabolic potential of soil microbial communities, future research integrating meta-transcriptomic approaches or enzymatic determinations will be essential to validate these inferred pathways and determine their actual in situ expression across different grazing management regimes.

## 5. Conclusions

The evaluated grazing-management regimes exhibited distinct soil carbon pools and bacterial community composition, while PICRUSt2 suggested possible differences in functional potential across the areas. While alpha diversity remained unaltered, grazing practices significantly influenced beta diversity shifts. Specifically, continuous grazing and rotational grazing at high stocking density corresponded with distinct shifts in bacterial community structures and biomarker distributions, such as the enrichment of copiotrophic taxa under managed grazing and oligotrophic groups under closed non-selective intensive grazing. Furthermore, predicted functional profiles indicated potential variations in metabolic pathways related to soil carbon and stress responses among the systems. Key pathways, such as tRNA charging and nicotinate degradation, may underscore potential microbial adaptations to heterogeneous, excreta-derived nutrient microsites during intermediate recovery states. However, these findings are based on a cross-sectional sampling design and predictive bioinformatic analyses, which inherently limit causal inferences and direct evidence of metabolic activity. Consequently, while these microbial indicators provide valuable insights into soil carbon stocks and bacteriome responses to grazing regimes, future research incorporating longitudinal monitoring, higher sample size, and meta-transcriptomic approaches remains essential to validate these predicted pathways. Finally, careful consideration of local management strategies remains vital for understanding how grazing practices influence soil microbial ecology in semi-arid ecosystems.

## Figures and Tables

**Figure 1:**
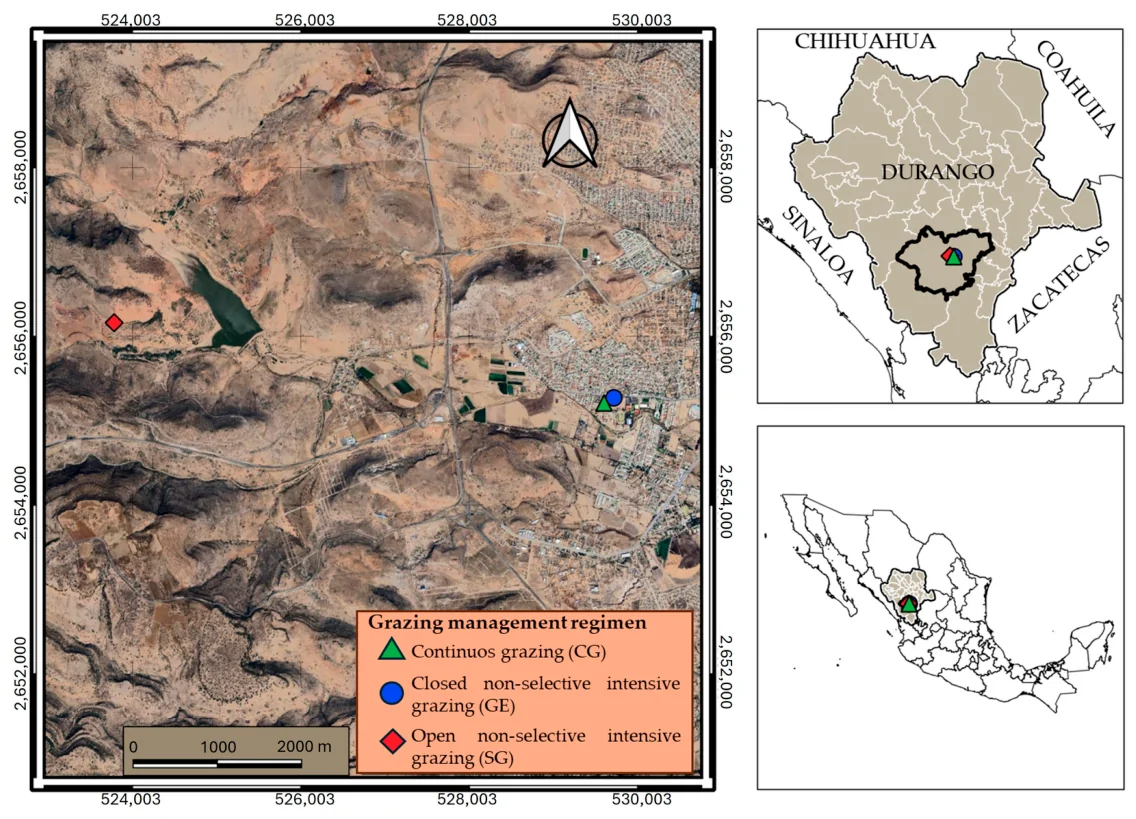
Geographic location of study areas in Lazaro Cardenas, Durango, Durango, Mexico.

**Figure 2:**
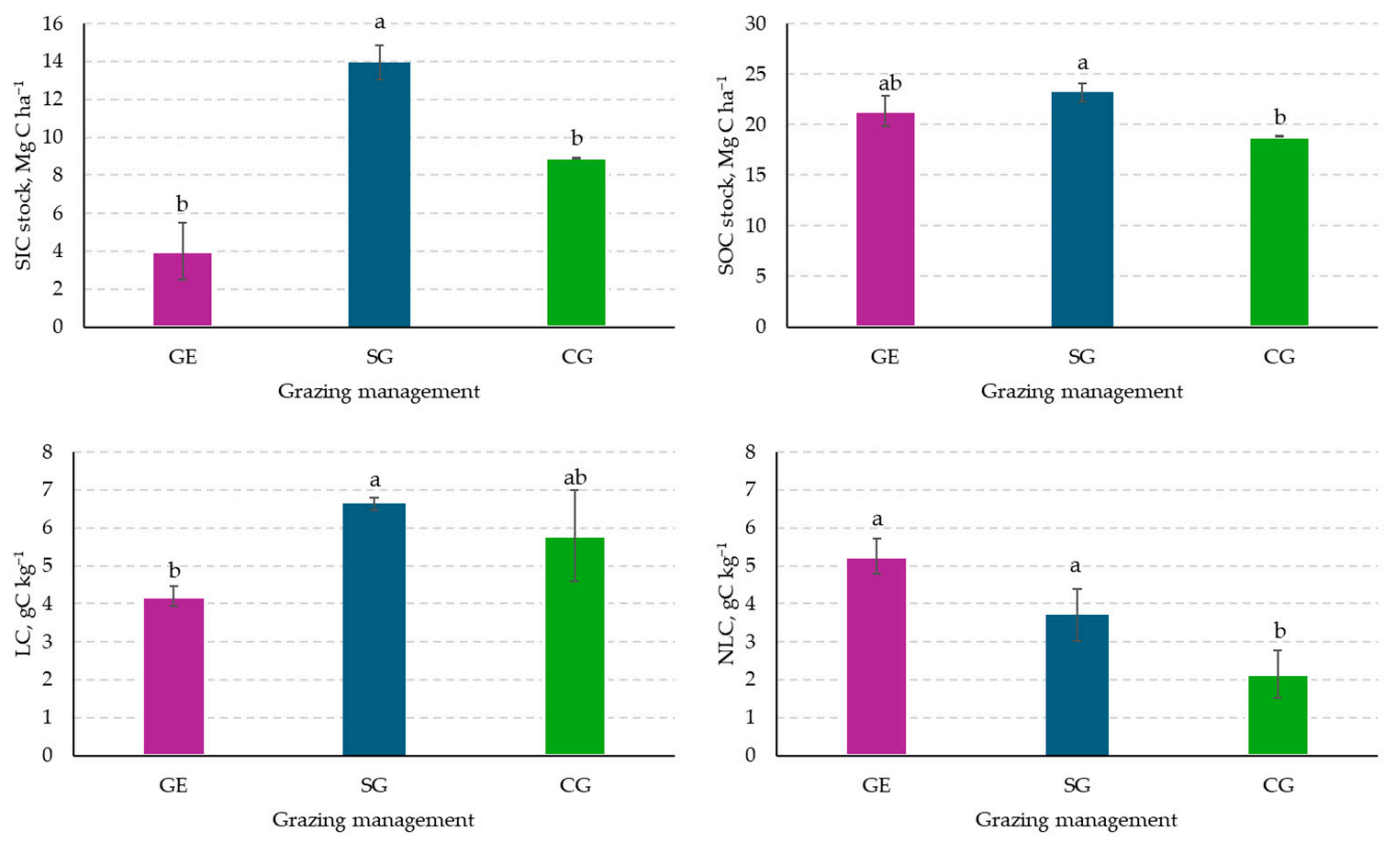
Soil organic and inorganic carbon stocks, along with labile and non-labile carbon fractions, across different grazing management regimes (*n* = 3 spatial replicates per treatment): GE—closed non-selective intensive grazing; SG—open non-selective intensive grazing; CG—continuous grazing; SIC stock—soil inorganic carbon stock; SOC stock—soil organic carbon stock; LC—labile carbon; NLC—non-labile carbon. Different lowercase letters above the bars indicate statistically significant differences among treatments according to Tukey’s test (*p* < 0.05).

**Figure 3:**
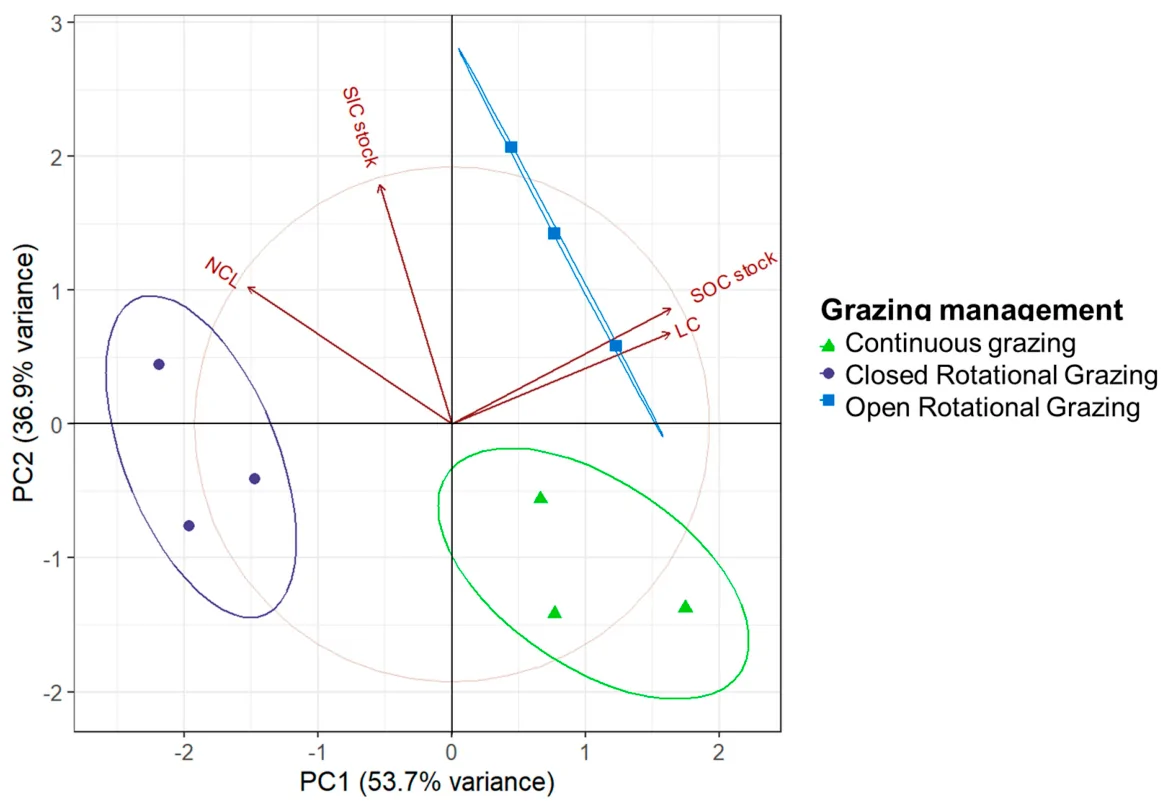
Principal Component Analysis (PCA) of soil carbon stocks and fractions of different lability under different grazing management (*n* = 3 spatial replicates per treatment): GE—closed non-selective intensive grazing; SG—open non-selective intensive grazing; CG—continuous grazing. SOC stock—soil organic carbon stock; SIC stock—soil inorganic carbon stock; LC—labile carbon fraction; NLC—non-labile carbon fraction. Solid colored ellipses represent group clustering or confidence regions for each grazing management treatment, while the larger faint circle represents the correlation circle of the variables.

**Figure 4:**
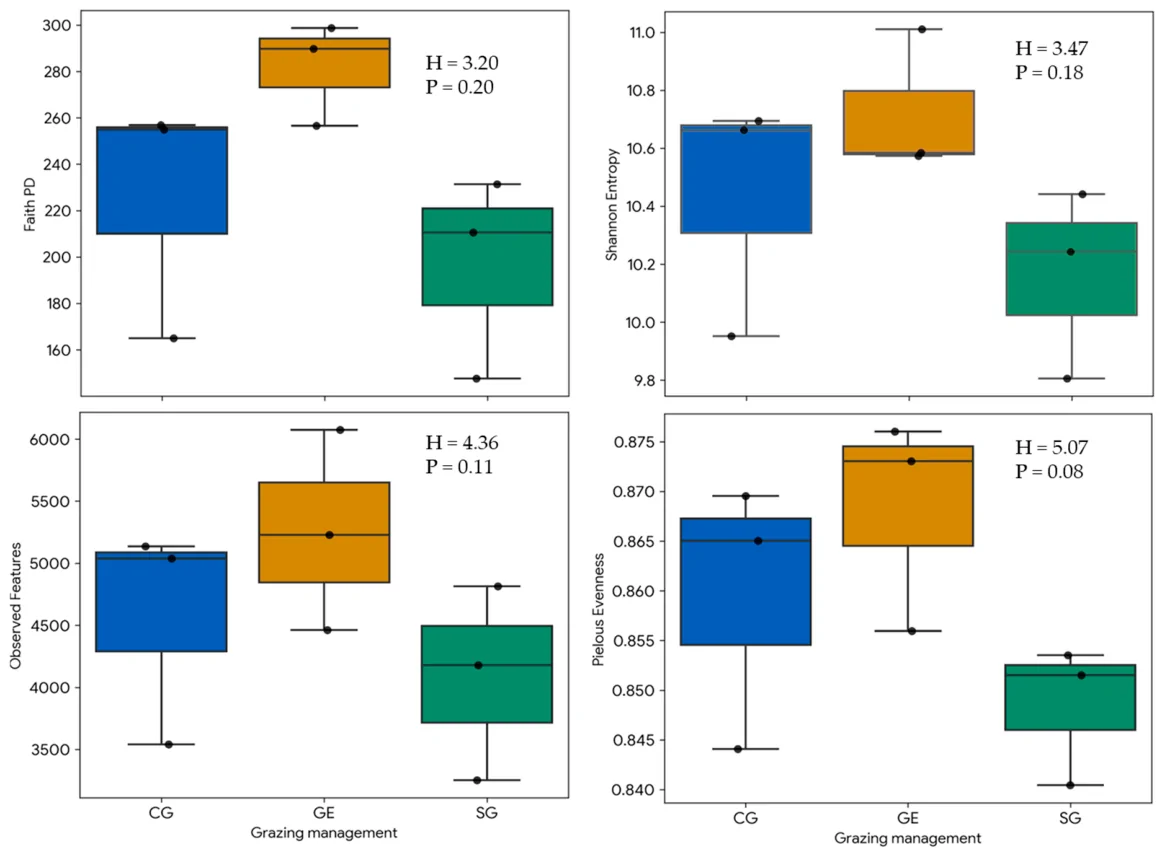
Alpha diversity indices (observed features [natural log-transformed], Shannon entropy, Faith’s phylogenetic Diversity, and Pielou’s evenness) across the evaluated grazing management systems (CG, GE, and SG). Boxplots display the median and interquartile ranges, with individual data points representing biological replicates (*n =* 3 spatial replicates per treatment). Kruskal–Wallis test statistics are indicated in each panel. GE—closed non-selective intensive grazing; SG—open non-selective intensive grazing; CG—continuous grazing.

**Figure 5:**
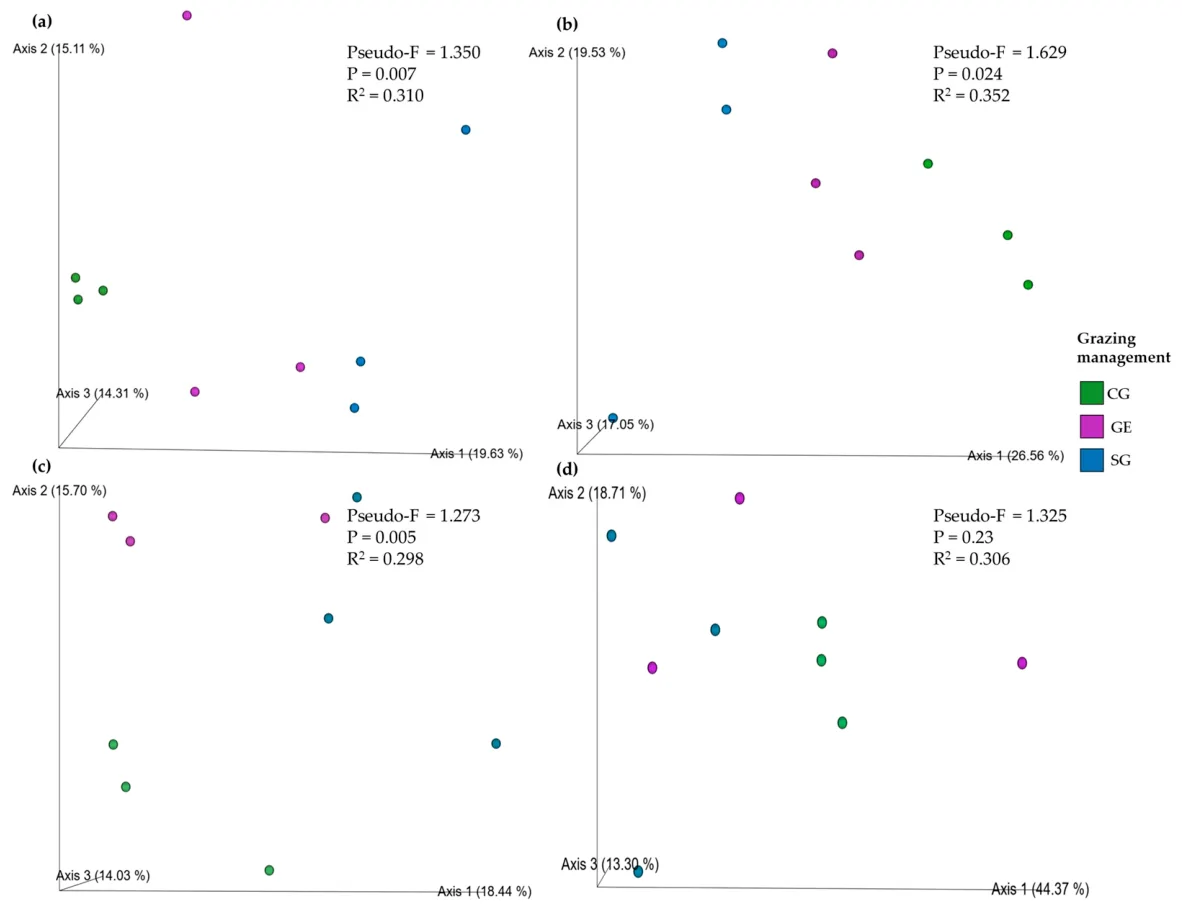
Beta diversity ordination of soil bacterial communities under different grazing management systems, evaluated via: (**a**) Jaccard distance; (**b**) Bray–Curtis dissimilarity; (**c**) unweighted UniFrac; and (**d**) weighted UniFrac matrices. Statistical significance was evaluated using PERMANOVA with 999 permutations (*p* < 0.05). GE—closed non-selective intensive grazing; SG—open non-selective intensive grazing; CG—continuous grazing.

**Figure 6:**
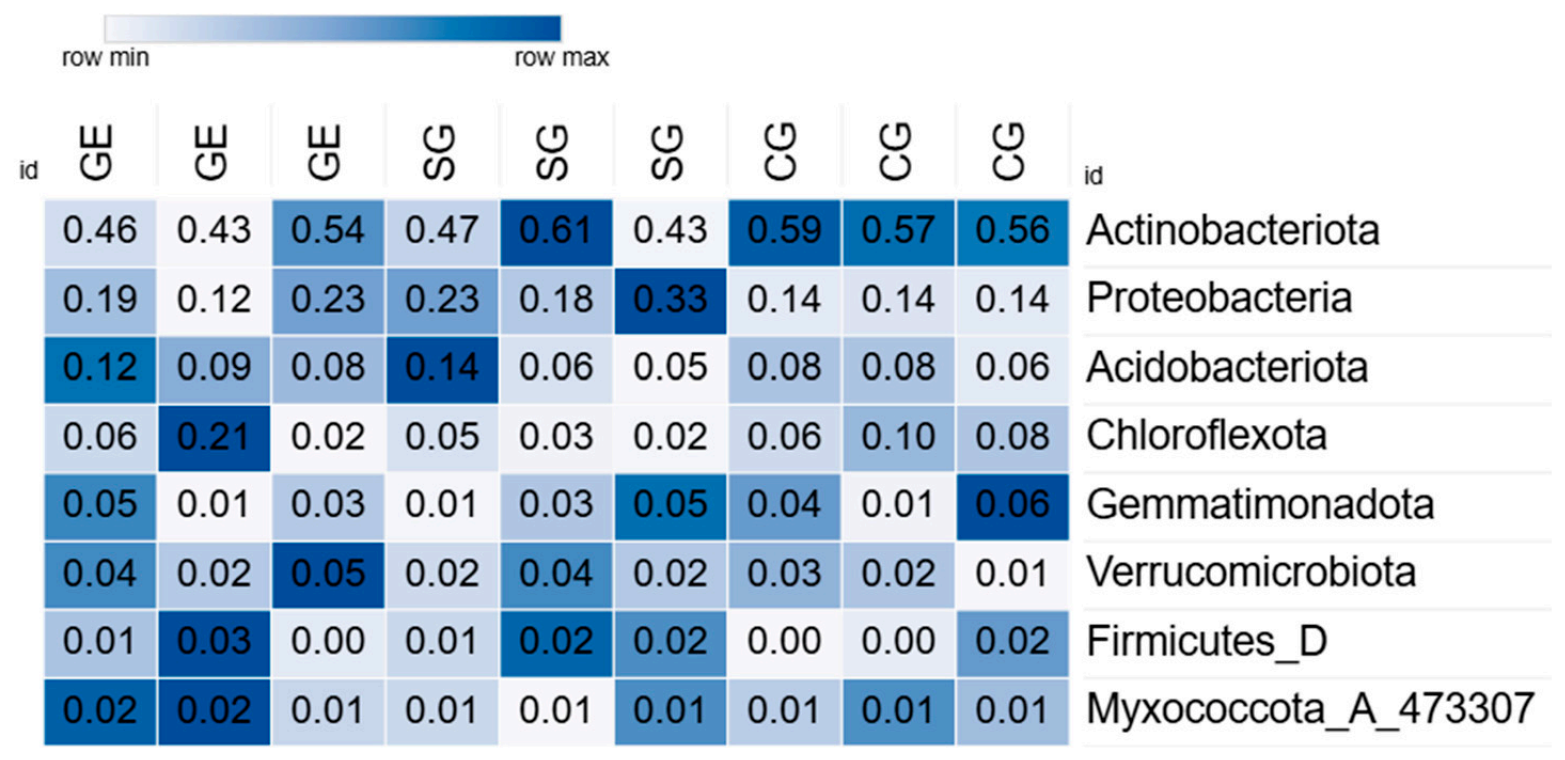
Relative abundance distribution of major bacterial phyla (restricted to taxa with a mean relative abundance > 1) across different grazing management regimes. Treatments include closed non-selective intensive grazing (GE), open non-selective intensive grazing (SG), and continuous grazing (CG).

**Figure 7:**
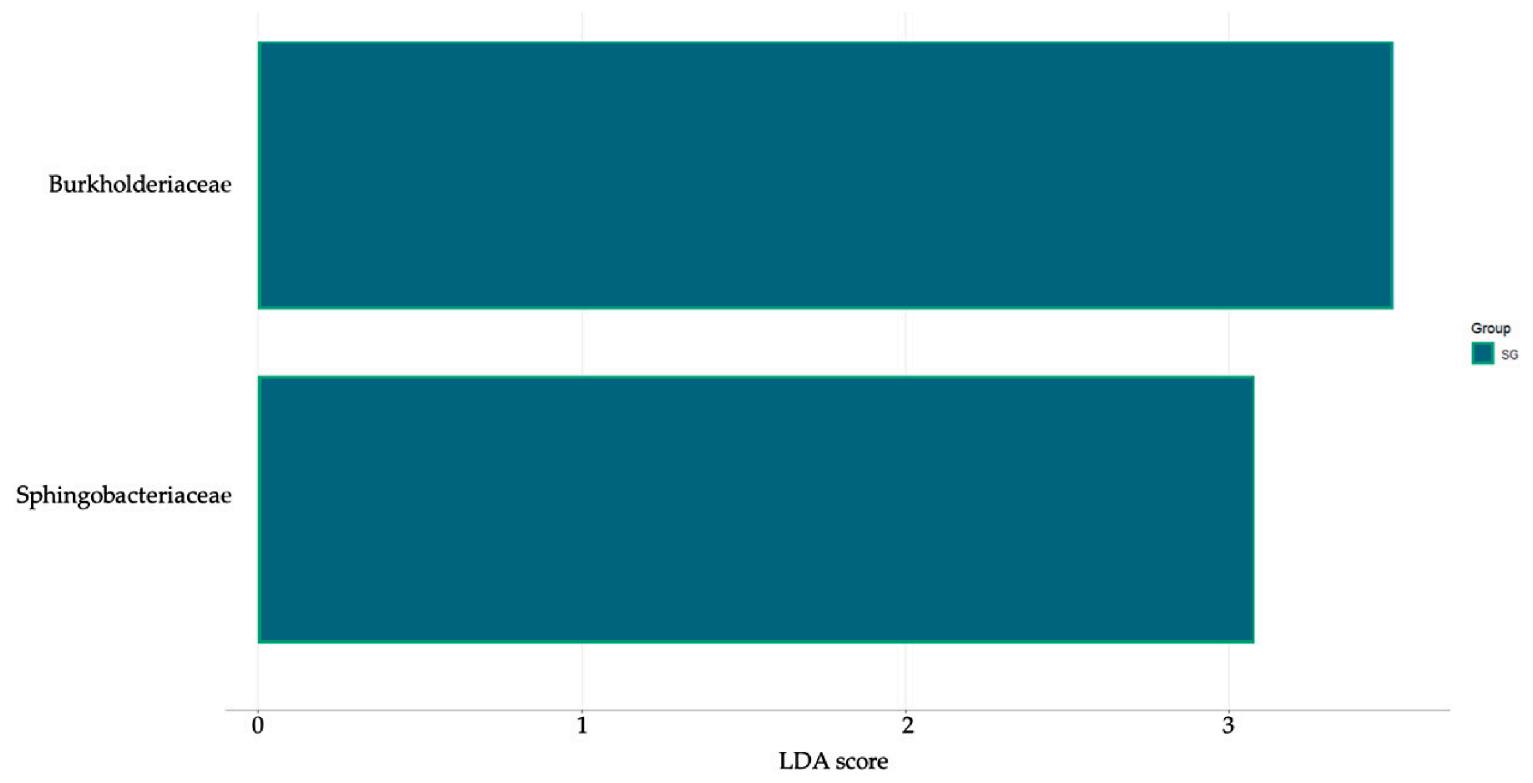
Linear Discriminant Analysis Effect Size (LEfSe) showing biomarker bacterial families enriched in open non-selective intensive grazing (SG) (*n =* 3 independent spatial replicates per treatment; threshold: LDA score > 3.0, *p* < 0.05, Kruskal–Wallis and pairwise Wilcoxon tests).

**Figure 8:**
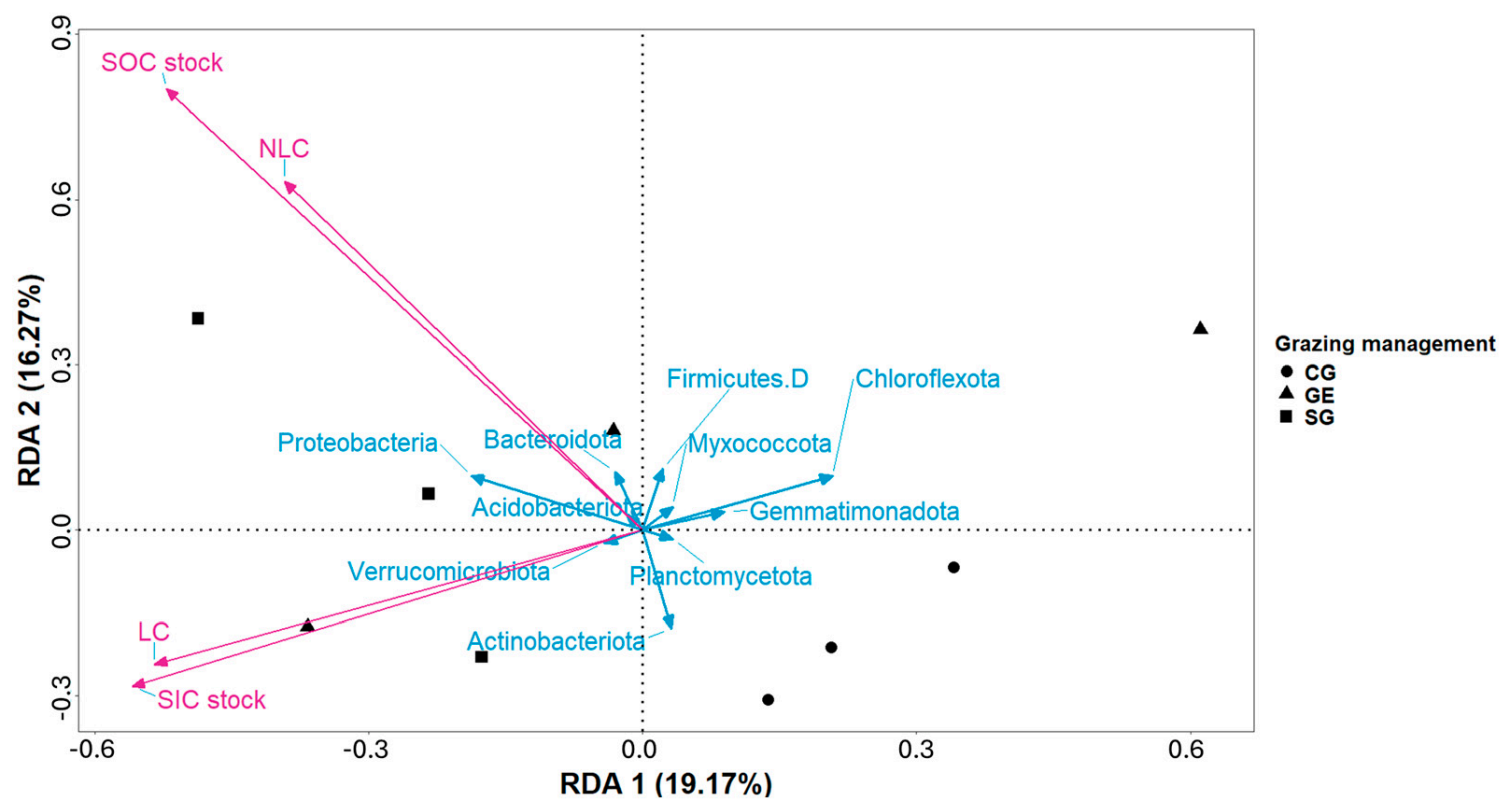
Redundancy Analysis relating bacterial phyla composition to soil carbon pools under different grazing management. The constrained model explained 47.88% of the total variance (global permutation test: F = 0.9185, *p* = 0.565, 999 permutations; residual degrees of freedom = 4). Variance inflation factors (VIFs) for all explanatory variables remained below 10 (ranging from 3.68 to 9.19). GE—closed non-selective intensive grazing; SG—open non-selective intensive grazing; CG—continuous grazing; SIC stock—soil inorganic carbon stock; SOC stock—soil organic carbon stock; LC—labile carbon; NLC—non-labile carbon.

**Figure 9:**
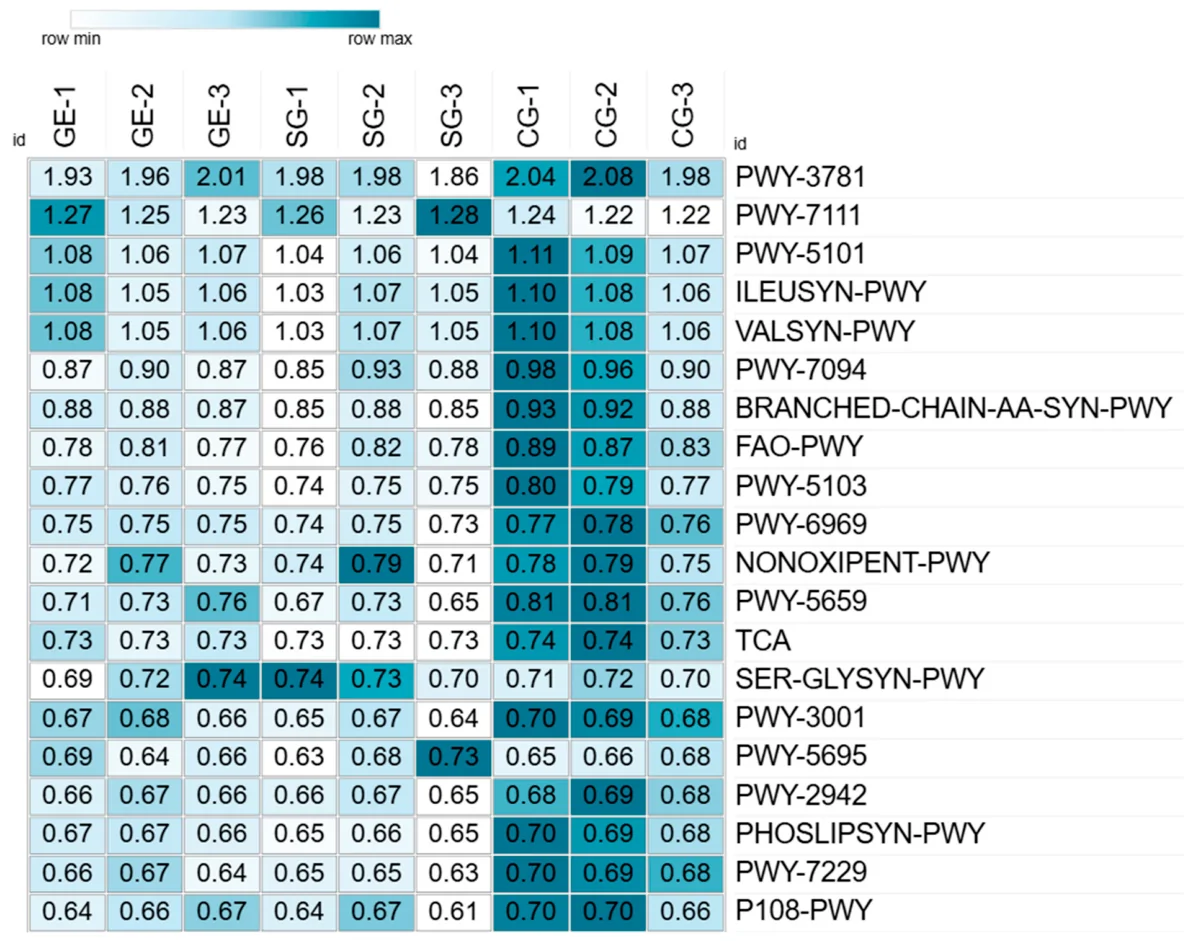
Relative abundance of the most abundant metabolic pathways inferred via PICRUSt2 from 16S rRNA gene sequencing data (*n =* 3 spatial replicates per treatment; ANOVA followed by Tukey’s HSD test, *p* < 0.05). GE—closed non-selective intensive grazing; SG—open non-selective intensive grazing; CG—continuous grazing.

**Figure 10:**
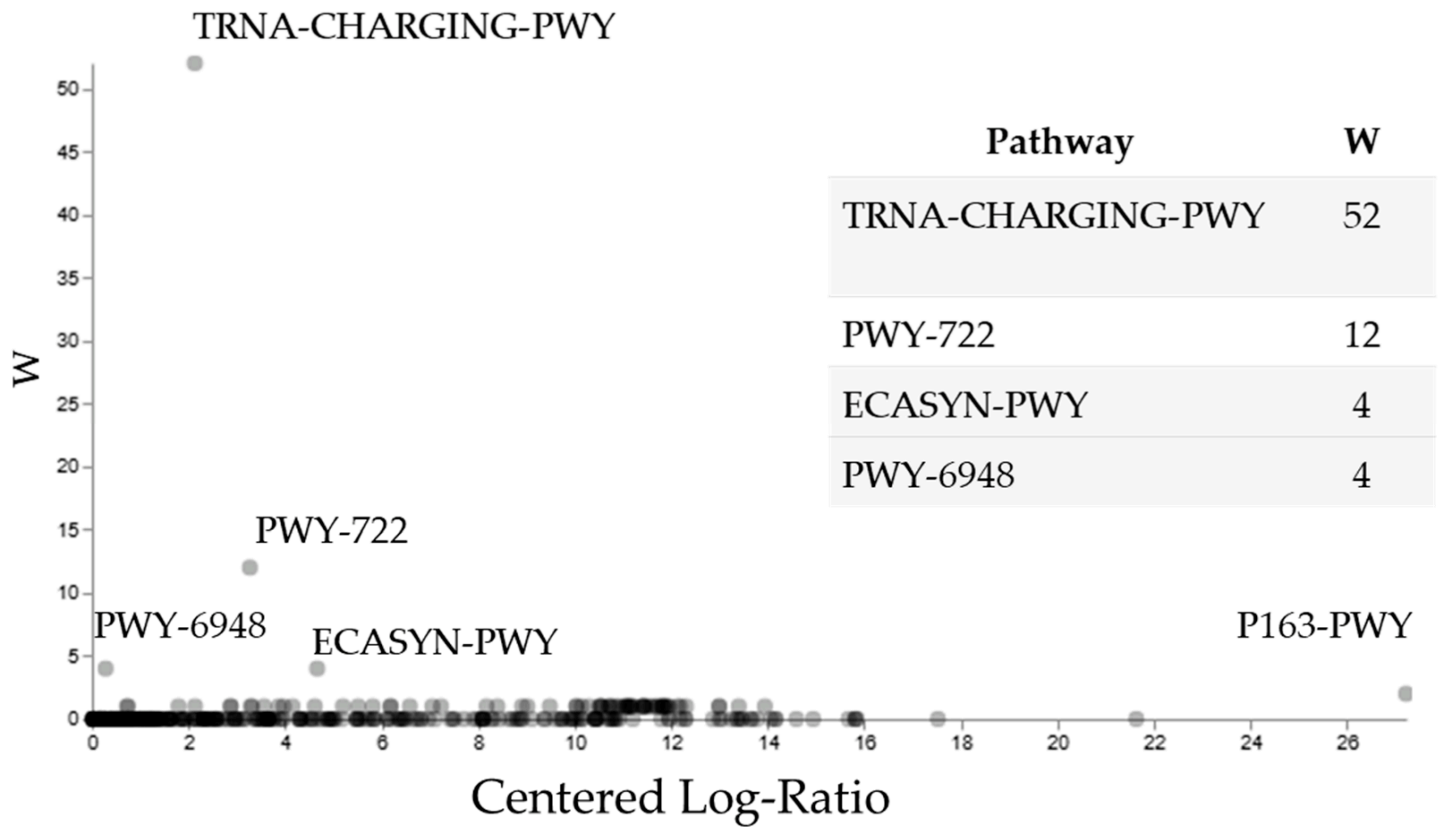
ANCOM of predicted metabolic pathways across grazing management systems. Significant functional pathways are highlighted based on their test statistic (W) (*n =* 3 spatial replicates per treatment; W ≥ 0.7 detection threshold).

## Data Availability

The following supporting information can be downloaded from the National Center for Biotechnology Information for public knowledge as BioProject: PRJNA1520797. The complete bioinformatic command-line workflows, script protocols, and analytical parameters for QIIME2 and PICRUSt2 analyses are openly available on GitHub at https://github.com/erinava27/QIIME2-Workflow-for-Soil-Bacterial-Community-Analysis.git (accessed on 15 October 2026).

## Supplementary Materials

The following supporting information can be downloaded at https://www.mdpi.com/article/10.3390/biology15201901/s1: Table S1. Baseline physicochemical properties in soils under different grazing management. Closed non-selective intensive grazing (GE); open non-selective intensive grazing (SG); continuous grazing (CG). Table S2. Summary of DADA2 denoising statistics, including input, filtered, denoised, and non-chimeric sequence counts, along with Weighted Nearest-Sequenced Taxon Index (NSTI) values for bacterial communities across the evaluated grazing management systems. Table S3. Analysis of Composition of Microbiomes (ANCOM) results highlighting predicted metabolic pathways with the strongest evidence of differential abundance (W statistics), along with the relative abundance of each pathway across 3 individual samples per treatment. Figure S1. Rarefaction curves depicting bacterial community richness across grazing management regimes: closed non-selective intensive grazing (GE), open non-selective intensive grazing (SG), and continuous grazing (CG). The curves represent the accumulation of observed amplicon sequence variants (ASVs) relative to sequencing depth. The observed plateaus suggest that the sequencing effort was sufficient to characterize the majority of the bacterial taxonomic diversity within the samples. Figure S2. Biomarker bacterial families identified by LEfSe showing significant enrichment across different grazing management regimes (Kruskal–Wallis test, *p* < 0.05, with an LDA score threshold > 2.0). CG: continuous grazing; GE: closed non-selective intensive grazing; SG: open non-selective intensive grazing. Figure S3. Relative abundance of the most dominant bacterial genera across different grazing management regimes: CG: continuous grazing; GE: closed non-selective intensive grazing; SG: open non-selective intensive grazing.
