# Supplementary material for: Grazing Management Regimen Shapes Soil Carbon Stocks and Associated Bacterial Community Composition in a Semi-Arid Rangeland
Source: Biology (Basel). 2026 Oct 21;15(20):1901. doi: 10.3390/biology15201901 (PMC13701752; doi:10.3390/biology15201901)
Supplement: Supplementary file 1 [file biology-15-01901-s001.zip › Figure S1.pdf]

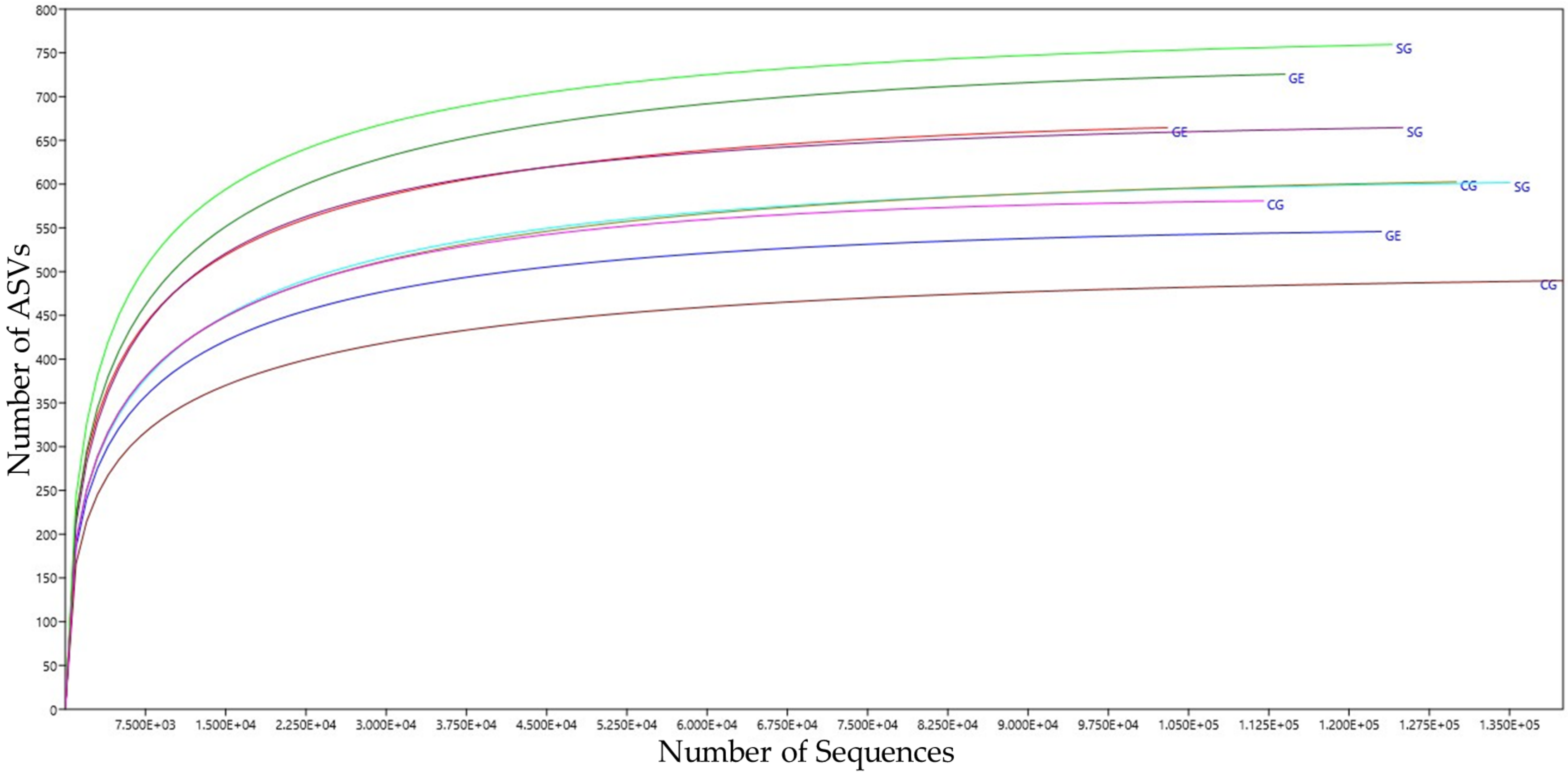

Figure S1. Rarefaction curves depicting bacterial community richness across grazing management regimes: closed non-selective intensive grazing (GE), open non-selective intensive grazing (SG), and continuous grazing (CG). The curves represent the accumulation of observed Amplicon Sequence Variants (ASVs) relative to sequencing depth. The observed plateaus suggest that the sequencing effort was sufficient to characterize the majority of the bacterial taxonomic diversity within the samples.
