# Supplementary material for: Grazing Management Regimen Shapes Soil Carbon Stocks and Associated Bacterial Community Composition in a Semi-Arid Rangeland
Source: Biology (Basel). 2026 Oct 21;15(20):1901. doi: 10.3390/biology15201901 (PMC13701752; doi:10.3390/biology15201901)
Supplement: Supplementary file 1 [file biology-15-01901-s001.zip › Figure S3.pdf]

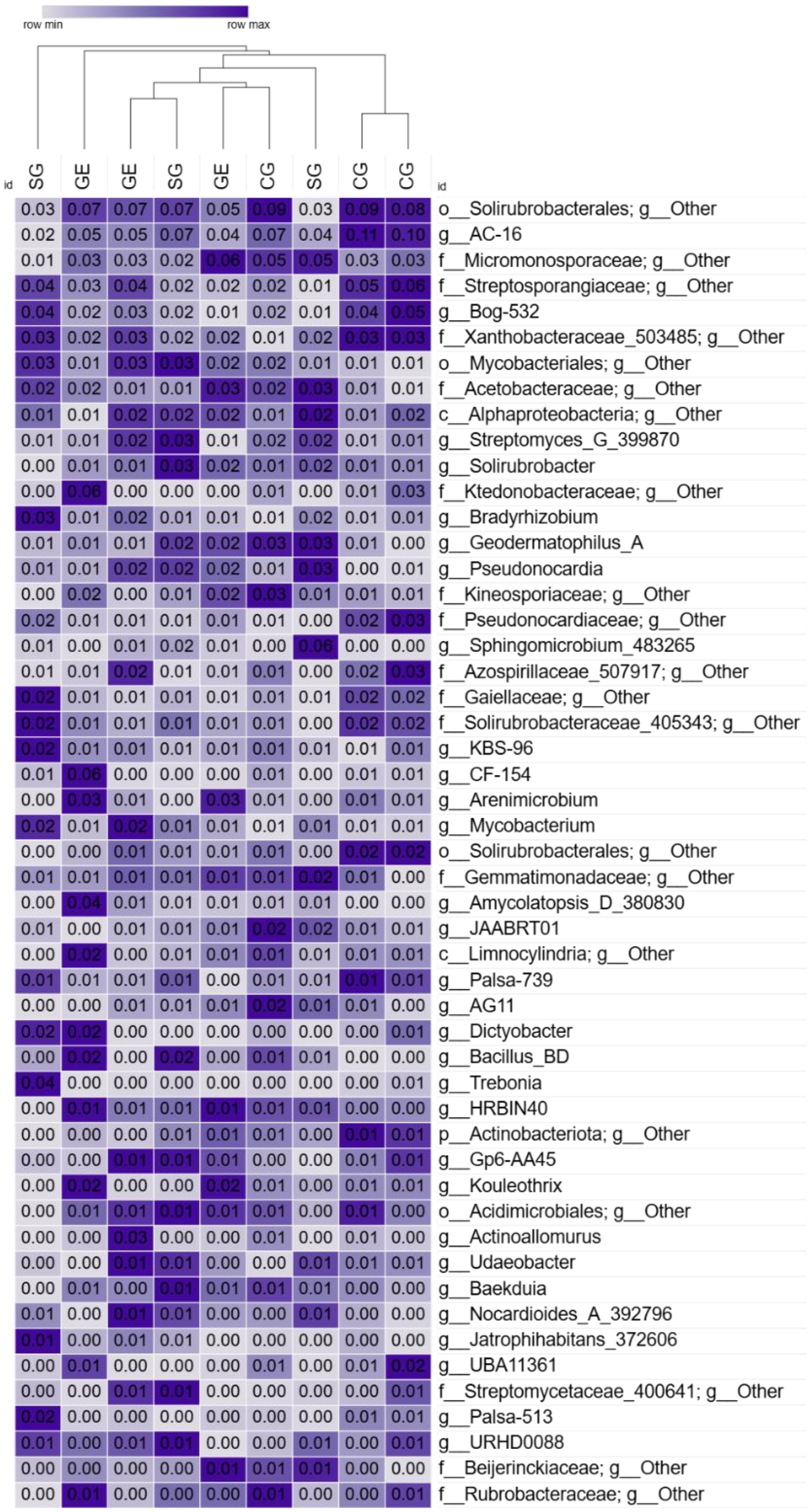


**Figure S3.** Relative abundance of the most dominant bacterial genera across different grazing management regimes: CG: continuous grazing; GE: closed non-selective intensive grazing; SG: open non-selective intensive grazing.
