# Supplementary material for: Grazing Management Regimen Shapes Soil Carbon Stocks and Associated Bacterial Community Composition in a Semi-Arid Rangeland
Source: Biology (Basel). 2026 Oct 21;15(20):1901. doi: 10.3390/biology15201901 (PMC13701752; doi:10.3390/biology15201901)
Supplement: Supplementary file 1 [file biology-15-01901-s001.zip › Table S1.pdf]

Table S1. Baseline physicochemical properties in soils under different grazing management. Closed non-selective intensive grazing (GE); Open non-selective intensive grazing (SG); Continuous grazing (CG).

Soil indicator	Closed Non-Selective Intensive Grazing	Continuous Grazing	Open Non-Selective Intensive Grazing
pH	6.20 ± 0.11	6.74 ±0.02	6.39 ±0.13
Electrical conductivity, dS m-1	0.20 ± 0.04	0.12 ± 0.01	0.14 ± 0.03
Bulk density, g cm-3	1.13±0.01	1.46 ± 0.02	1.20 ± 0.02
C:N	6.64 ± 0.30	3.52 ± 0.11	5.49 ± 0.10
Texture	Sandy loam	Sandy loam	Sandy loam
