# Supplementary material for: Grazing Management Regimen Shapes Soil Carbon Stocks and Associated Bacterial Community Composition in a Semi-Arid Rangeland
Source: Biology (Basel). 2026 Oct 21;15(20):1901. doi: 10.3390/biology15201901 (PMC13701752; doi:10.3390/biology15201901)
Supplement: Supplementary file 1 [file biology-15-01901-s001.zip › Table S2.pdf]

Table S2. Summary of DADA2 denoising statistics, including input, filtered, denoised, and non-chimeric sequence counts, along with Weighted Nearest-Sequenced Taxon Index (NSTI) values for bacterial communities across the evaluated grazing management systems.

Sample-id	Input	Filtered	Percentage of input passed filter	Denoised	Merged	Percentage of input merged	Non-chimeric	Percentage of input non-chimeric	Weighted NSTI[1]
GE-1	205890	196391	95.39	188440	141488	68.72	104840	50.92	0.142
GE-2	205950	196695	95.51	190933	163021	79.16	125583	60.98	0.145
GE-3	210123	200850	95.59	194879	155475	73.99	116547	55.47	0.138
SG-1	202703	194323	95.87	190951	169457	83.6	136997	67.59	0.135
SG-2	203540	194802	95.71	190614	165889	81.5	128942	63.35	0.133
SG-3	204370	195744	95.78	190563	163212	79.86	126589	61.94	0.108
CG-1	217219	207622	95.58	202225	169648	78.1	131804	60.68	0.167
CG-2	206740	197267	95.42	193764	170160	82.31	141760	68.57	0.169
CG-3	206461	197779	95.79	191649	149763	72.54	113771	55.11	0.153

[1]The weighted NSTI values measure the mean phylogenetic distance separating sample ASVs from reference genomes, thereby indicating the reliability of the predicted metabolic functions.
