# Supplementary material for: Grazing Management Regimen Shapes Soil Carbon Stocks and Associated Bacterial Community Composition in a Semi-Arid Rangeland
Source: Biology (Basel). 2026 Oct 21;15(20):1901. doi: 10.3390/biology15201901 (PMC13701752; doi:10.3390/biology15201901)
Supplement: Supplementary file 1 [file biology-15-01901-s001.zip › Table S3.pdf]

Table S3. Analysis of Composition of Microbiomes (ANCOM) results highlighting predicted metabolic pathways with the strongest evidence of differential abundance (W statistics), along with the relative abundance of each pathway across 3 individual simples per treatment.

Pathway	Description	W	GE-1	GE-2	GE-3	SG-1	SG-2	SG-3	CG-1	CG-2	CG-3
TRNA-CHARGING-PWY	tRNA charging	52	0.368	0.346	0.353	0.362	0.349	0.360	0.298	0.286	0.287
PWY-722	Nicotinate degradation I	12	0.015	0.019	0.018	0.028	0.027	0.020	0.010	0.012	0.008
ECASYN-PWY	Enterobacterial common antigen biosynthesis	4	$8.70X10^{-6}$	0.000	0.000	$6.58X10^{-6}$	$5.08\ X10^{-5}$	$5.01\ X10^{-5}$	0.000	0.000	0.000
PWY-6948	Sitosterol degradation to androstenedione	4	0.013	0.012	0.022	0.021	0.015	0.014	0.011	0.011	0.009
P163-PWY	L-lysine fermentation to acetate and butanoate	2	0.134	0.116	0.117	0.069	0.081	0.093	0.080	0.084	0.123

* GE - Closed rotational grazing; SG - Open rotational grazing; CG - Continuous grazing. ANCOM models the compositional data to identify features that are differentially abundant across multiple pairwise treatment comparisons. The W statistic represents the number of times the null hypothesis of no differential abundance was rejected for a given metabolic pathway relative to all other tested features. Higher W values indicate stronger statistical support for differential enrichment among the evaluated grazing regimes.
